# Combinatorial Interactions of Essential Oils Enriched with Individual Polyphenols, Polyphenol Mixes, and Plant Extracts: Multi-Antioxidant Systems

**DOI:** 10.3390/antiox12020486

**Published:** 2023-02-15

**Authors:** Marina Minh Nguyen, Salwa Karboune

**Affiliations:** Department of Food Science and Agricultural Chemistry, Macdonald Campus, McGill University, Ste Anne de Bellevue, QC H9X3V9, Canada

**Keywords:** antioxidants, essential oils, polyphenols, plant extracts, synergy, multi-antioxidant systems

## Abstract

With the aim to develop essential oil (EO) multi-antioxidant systems, combinatorial interactions of selected phenol and terpene-rich EOs (from Pimento Berry, Ceylon Cinnamon, Clove, Sage, White thyme; Oregano) enriched with individual polyphenols, crude plant extracts, and mixtures of their major polyphenols were investigated using single electron transfer (SET)-based DPPH and hydrogen atom transfer (HAT)-based ORAC assays. Polyphenols that enriched Eos the most favorably were rosmarinic acid (IC_50_ of 0.0891–0.1448 mg enriched EO/mg DPPH; 5772–17,879 µmol TE/g enriched EO) and quercetin (IC_50_ of 0.0682–0.1060 mg enriched EO/mg DPPH; Trolox Equivalents (TE) of 9776–14,567µmol /g enriched EO), whereas *p*-coumaric acid (IC_50_ of 0.1865–1.1424 mg enriched EO/mg DPPH; 7451.00–11,588 µmol TE/g enriched EO) and rutin hydrate (IC_50_ of 0.1140–0.3112 mg enriched EO/mg DPPH; 2298–6227 µmol TE/g enriched EO) were the least favorable. Enrichments with polyphenol mixes and crude extracts exhibited synergistic and additive effects in the SET-based DPPH assay. In the HAT-based ORAC assay, EO enrichments with crude extracts exhibited more additive effects, as well as less antagonistic effects, than enrichments with their major polyphenol mixes, revealing the significant contributions of minor compounds. EOs enriched with crude green tea and apple extracts exhibited synergistic or additive effects, whereas EOs enriched with grape seed and rosemary extracts exhibited equal antagonistic effects. Predictive models were developed to explain the variability between the observed and predicted antioxidant activities of enriched EOs.

## 1. Introduction

To limit the oxidative degradation of lipids during storage and distribution, the food industry has employed the use of synthetic antioxidants, which have since fallen under scrutiny. Indeed, synthetic antioxidants, such as butylated hydroxyanisole, butylated hydroxytoluene, tert-butylhydroquinone, and propyl gallate, are suspected to have carcinogenic, mutagenic, and teratogenic effects after chronic use [1]. With increasing concerns regarding the safety of these preservatives as well as increasing consumer demands for more natural products, the industry is now considering essential oils (EOs) and plant extracts as potential natural replacements. These alternatives are naturally occurring phytochemicals that have shown a wide range of biological activities and physicochemical properties. Indeed, several studies have documented the antioxidant and antimicrobial activities of EOs, plant extracts and their components [1,2].

EOs are composed of volatile compounds that contribute to their antimicrobial and antioxidant properties such as phenols, allylic alcohols, and selected monoterpenes [3,4]. The chemical profile of EOs is often dominated by two or three major compounds, but their antioxidant activity may also rely on synergistic interactions in situ with the EO’s minor components [5]. In our previous study, correlation analysis of the chemical profiles of 38 EOs showed concurrent abundance of some compounds; for instance, monoterpenes and alcohols were concurrently present, and phenol-rich EOs had a higher content of sesquiterpenes [6]. The antioxidant activity of phenols paired with esters and alcohols showed synergistic effects when measured with the ORAC assay, which is a hydrogen atom transfer-based reaction. When phenols were paired with monoterpenes and ketones, antagonistic effects were observed when measuring the antioxidant activity with the DPPH Assay, whose reaction is driven by single electron transfer [6]. 

Blends of antioxidants can have an additive, synergistic, antagonistic, or indifferent effect [5,7]. In an additive effect, the sum of the individual components will be equal to the combined activity of the blend of antioxidants. When the combined effect of the blend of oils is greater than the sum of their individual components, the effects are synergistic. When antagonism occurs, the combined effect is less than the sum of their individual components. In the present study, combinations of EOs with individual major polyphenols, polyphenols mixes, or crude plant extracts were assessed for their antioxidant activities and combinatorial effects. To the best of our knowledge, no study so far has explored the interactive effects between these combinations. The EOs included pimento berry (HE-PIM-01), clove (HE-CLO-01), oregano (HE-ORI-03), white thyme (HE-THY-03), yellow sage (HE-SAU-01-02), and Ceylon cinnamon (HE-CAN-04), while the extracts included grape seed (EX-RAI-01), green tea (HE-THE-01), apple (EX-POM-04), and rosemary (EX-ROM-04). Two methods utilizing the abilities to jointly transfer electrons or hydrogen atoms were used—the DPPH (2,2′-diphenylpicryl hydrazyl free radical) assay and ORAC (Oxygen Radical Absorbance Capacity) assay. The characterization of interactions between EOs and individual major polyphenols, polyphenols mixes, or crude plant extracts will enable the development of efficient multi-antioxidant systems.

## 2. Materials and Methods

### 2.1. Materials

EOs were obtained from commercial suppliers (Table S1). Grape seed and green tea extracts were obtained from New Directions (Mississauga, ON, Canada). Apple extract was obtained from Diana Food (Champlain, QC, Canada). Rosemary extract was obtained from BSA (Saint-Léonard, QC, Canada). DPPH (>90%), α-tocopherol (96%), 2,2′-Azobis(2-methylpropionamidine) dihydrochloride (AAPH), fluorescein sodium salt, and 6-hydroxy-2,5,7,8-tetramethylchroman-2-carboxylic acid (Trolox) were purchased from MilliporeSigma (Burlington, MA, USA). Acetone (≥99.5%) and methanol (≥99.9%) were purchased from Thermo Fisher Scientific (Waltham, MA, USA). Anhydrous ethanol (≥99.9%) was purchased from Commercial Alcohols Inc. (Brampton, ON, Canada).

### 2.2. Preparation of Antioxidants

EOs and their enriched counterparts were prepared according to their compositions (Tables S1 and S2). The chemical profile of EOs was characterised in our previous study [6], and the major families of EOs compounds are shown in Table S1. The major polyphenol mixes were prepared in ethanol in proportions present in grapeseed (48% *w*/*w* catechin, 52% *w*/*w* epicatechin), green tea (1.1% *w*/*w* chlorogenic acid, 21% *w*/*w* catechin, 76% *w*/*w* epicatechin, 0.4% *w*/*w p*-coumaric acid, 1.2% *w*/*w* rutin hydrate), apple (81% *w*/*w* chlorogenic acid, 2.1% *w*/*w* epicatechin, 4.3% *w*/*w* rutin hydrate, 12% *w*/*w* quercetin), and rosemary (80% *w*/*w p*-coumaric acid, 20% *w*/*w* rosmarinic acid) extracts to simulate them (Table S2). All EOs were enriched with individual major polyphenols and polyphenol mixes at a ratio of 1:1 (*w*/*w*). EOs and plant extracts were mixed to achieve the same ratio as EOs and polyphenol mixes.

### 2.3. 2,2-Diphenyl-1-Picrylhydrazyl (DPPH) Radical Scavenging Assay

The DPPH assay was carried out according to the method reported by Brand-William et al. [8] with modifications. Varying concentrations of antioxidants were added to Tris-HCl buffer (450 µL, pH 7.4). DPPH (0.1 mM) was then added to all samples and incubated in the dark for 30 min at room temperature. In preliminary trials, 30 min was identified as the appropriate time at which the steady state of scavenging was achieved. Then, the absorbance was read at 517 nm using a Beckman spectrophotometer. Water was used as the blank. As the negative control, 10% methanol in Tris-HCl buffer was used. α-tocopherol in ethanol (2 mg/mL) was used as the positive control. The inhibition ratio (%) was calculated as follows:

Inhibition ratio (%) = [(Ac − As)/Ac] × 100(1)

where Ac is the absorbance of the control; As is the absorbance of the sample.

The concentrations of antioxidants were plotted against the estimated inhibition ratio. The IC_50_ was defined as the concentration required to reduce DPPH by 50%. The IC_50_ was expressed as mg sample/mg DPPH.

### 2.4. Oxygen Radical Absorption Capacity (ORAC) Assay

The ORAC assay was carried out according to the method outlined by [9], with modifications. AAPH (2,2′-azobis(2-amidinopropane) dihydrochloride) was used as a free-radical generator and varying concentrations of selected essential oils were used to prevent the decay of Fluorescein Sodium Salt. Trolox (±)-6-Hydroxy-2,5,7,8-tetramethylchromane-2-carboxylic acid (100, 50, 25, 12.5, 6.25, and 0 mM) was used as a standard and phosphate buffer (75 mM, pH 7.4) was used as a blank. Antioxidants prepared in acetone/phosphate buffer (pH 7.4) and 16 mM fluorescein (prepared in PBS 10 mM pH 7.4) were pipetted into a 96-well black-walled plate. Following incubation at 37 °C for 30 min in a Synergy HTX Multi-Mode Reader, all wells were injected with freshly prepared AAPH (79.65 mmol/L). Fluorescence readings were taken for 1 h at 485 nm (excitation wavelength) and 520 nm (emission wavelength) every 60 s. The AUC (area under the curve) and Net AUC of the standards and samples were determined using Gen5 Data Analysis Software using the following equations respectively:

(2) $$AUC=\left(\frac{R_{1}}{R_{1}}\right)+\left(\frac{R_{2}}{R_{1}}\right)+\left(\frac{R_{3}}{R_{1}}\right)+\ldots +\left(\frac{R_{n}}{R_{1}}\right)$$

where *R*_1_ is the fluorescence reading at the initiation of the reaction; *R_n_* is the last measurement

Net AUC = AUC_sample_ − AUC_blank_ (3)

The standard curve consisted of the Net AUC of various Trolox concentrations plotted against their concentration. Finally, to calculate the Trolox Equivalents (TE) of each sample range, the following equation was used:

TE (range of concentrations) = m_compound_/m_Trolox_ (4)

where m_compound_ is the slope of the linear regression analysis of the compound; m_Trolox_ is the slope of the linear regression analysis of Trolox.

Results are expressed as µmol Trolox Equivalents (TE)/g sample.

### 2.5. Statistical Analysis 

All measurements were taken as triplicates and reported as mean ± standard error. The statistical analyses were carried out using the Microsoft Excel 2019 software package (Microsoft Corp., Redmond, WA, USA). The Kruskal–Wallis and Dunn’s multiple comparisons tests were performed to detect significant differences (*p* < 0.05) using GraphPad Prism Version 8.4.2 for Windows, GraphPad Software, San Diego, CA, USA, www.graphpad.com. 

The antioxidant activity of the combinations was compared to their respective simulations. Synergistic, additive, and antagonistic effects were defined to have taken place if the measured antioxidant capacity was greater than, equal to, or less than the sum of the antioxidant capacity of the individual compounds in equimolar concentrations, respectively.

Antioxidant samples were clustered according to their IC_50_ and ORAC values and to their types using Ward’s method and heat map representations of variables were generated. Multivariate statistical analysis, including principal component analysis (PCA), was completed using R software (version 3.4). R software was also used to establish correlations between variables and responses. Design Expert^®^ Software version 8.0.7 (Stat-Ease, Inc., Minneapolis, MN, USA) was used to identify the best models that fit the relationships between the composition and the antioxidant properties of essential oils and for analysis of variance (ANOVA).

## 3. Results and Discussion

### 3.1. Antioxidant Capacity of EOs Enriched with Individual Polyphenols

The addition of singular major polyphenols of plant extracts to 6 EOs improved some of the latter’s antioxidant capacities but not most of the former (Table 1). These major compounds included four flavonoids (rutin hydrate, quercetin, epicatechin, and catechin) and four phenolic acids (rosmarinic, *p*-coumaric, chlorogenic, and ferulic acids). Regardless of the interactions with EOs, the IC_50_ and ORAC values of the enrichments followed the structural advantages of their respective phenolic acid or flavonoid enrichment. The average antioxidant activities of the EOs were as follows according to their phenolic acid or flavonoid enrichment, respectively: rosmarinic acid > chlorogenic acid > ferulic acid > *p*-coumaric acid, and quercetin > epicatechin > catechin > rutin hydrate. Phenolic acids and flavonoids are hydrogen-donating radical scavengers and their efficiencies increase with increasing hydroxyl groups in their aromatic moieties and catechol groups, respectively [7].

Ferulic acid and *p*-coumaric acid’s activities differed due to the methoxy substitution in the ortho position to the hydroxyl group [10]. Quercetin held the best flavonoid advantage thanks to the catechol in its B-ring and double bond and 4-oxo function in its C-ring. This unsaturation allows electron delocalization, which explains epicatechin and catechin’s lowered antioxidant capacity due to their saturated heterocyclic ring [10]. These two differed in activity due to the stereo-position of catechin’s 3-OH group in its C-ring [7]. Furthermore, glycosylation of quercetin at the 3-OH group, producing rutin, decreases its activity [11], which can be seen in the EO/rutin hydrate enrichments (Table 1). 

Due to combinatorial effects, the EO/polyphenol pair with the lowest IC_50_ was Ceylon cinnamon (HE-CAN-04)/quercetin (0.068 mg/mg DPPH), whereas yellow sage (HE-SAU-01-02)/*p*-coumaric acid had the highest IC_50_ (1.142 mg/mg DPPH). The former’s high antioxidant capacity is supported by the fact that quercetin can be found in situ in *Cinnamomum* species [12]. Therefore, the supplementary quercetin reinforces the oil’s pre-existing synergy. The latter pair’s low capacity could be due to the lack of phenols in the EO (Table S1), in addition to *p*-coumaric acid’s poor performance in the DPPH assay [13] due to the lack of a catechol group.

Mixtures enriched with rutin hydrate, quercetin, and *p*-coumaric acid exhibited additive and synergistic effects, and no antagonistic effects in the DPPH assay. Rutin hydrate has exhibited synergistic effects with terpenoids, such as γ-terpinene [14], which could explain its interactions with EOs. Interestingly, many pimento berry EO (HE-PIM-01) enrichments exhibited antagonistic effects in the DPPH assay, including those with rosmarinic acid (0.097 mg/mg DPPH), chlorogenic acid (0.134 mg/mg DPPH), catechin (0.088 mg/mg DPPH), and ferulic acid (0.367 mg/mg DPPH).

The EO/polyphenol pair with the highest ORAC value was white thyme (HE-THY-03)/ferulic acid (19,273.5 µmol TE/g oil + polyphenol), whereas clove (HE-CLO-01)/epicatechin (2252.4 µmol TE/g oil + polyphenol) displayed the lowest. There was no antagonism in enrichments with quercetin and catechin. Rutin hydrate enrichments exhibited the most antagonistic effects in the ORAC assay. Of the EOs, yellow sage (HE-SAU-01-02) exhibited the most antagonism when enriched with polyphenols, notably rutin hydrate (2298.5 µmol TE/g oil + polyphenol), rosmarinic acid (5772.0 µmol TE/g oil + polyphenol), *p*-coumaric acid (7451.0 µmol TE/g oil + polyphenol), and chlorogenic acid (4709.4 µmol TE/g oil + polyphenol). 

The synergy between certain EOs/polyphenols could be due to the regeneration of their antioxidants, where weaker antioxidants (co-antioxidant/synergist) regenerate stronger antioxidants (primary antioxidant), and vice versa in antagonism [7]. This synergy occurs when the primary antioxidant has a higher reduction potential than the synergist [15]. Quercetin, epicatechin, and catechin have regenerated α-tocopherol [15], thereby resulting in a co-antioxidant effect. However, other factors also contribute to the combinatorial effects in multi-component systems, including molecule polarity [16] and the influences of the microenvironment [17].

### 3.2. Antioxidant Capacity of EOs Enriched with Polyphenol Mixes

Aside from rosemary’s polyphenol mix, the IC_50_ of all unenriched polyphenol mixes were lower than their expected IC_50_, thus displaying antagonistic effects (Table 2). This could be due to flavonoid-flavonoid interactions, where H-bonding between flavonoids results in a decrease in the availability of -OH groups [18]. This, in turn, decreases the possibility of interacting with the DPPH radical, lowering the resulting antioxidant capacity. In the ORAC assay, all polyphenol mixes, except those with grape seed, showed synergistic effects. The ORAC value of a pair of flavonoids has been reported to significantly increase when a third flavonoid with a low reduction potential energy is added [19]. Additionally, mixtures of compounds with similar reduction potential energies have shown lower antioxidant activities [20] since the compounds drew electrons away without readily donating them to the AAPH radical. This could account for the antagonism within the grape seed mix. Although the ORAC assay is based on the transfer of a hydrogen atom, an electron is also transferred, which justifies the importance of analyzing the compounds’ reduction potentials.

All EO/grape seed mix enrichments showed synergistic effects in the DPPH assay (Table 3), except pimento berry (HE-PIM-01, 0.074 mg/mg DPPH), which showed additive effects. Similarly, all enrichments showed synergistic effects in the ORAC assay (Table 3), except that of oregano (HE-ORI-03, 1219.40 μmol TE/g sample), which exhibited antagonistic effects. All EOs containing eugenol (clove, HE-CLO-01; pimento berry, HE-PIM-01; Ceylon cinnamon, HE-CAN-01) showed similar IC_50_ and ORAC values, whereas those lacking it (Table S1) exhibited lower ORAC values. This suggests that the mixture’s epicatechin and catechin interacted with the eugenol present in the EOs. As previously mentioned, oregano (HE-ORI-03)/grape seed mix showed antagonistic effects in the ORAC assay, whereas all other enrichments exhibited synergistic effects. Therefore, despite the antagonistic effects within the grape seed polyphenol mix in situ (Table 2), enriching it with EOs (Table 3), except oregano (HE-ORI-03), overcomes the antagonism in the ORAC assay. Perhaps this antagonism arises from the large quantity of α-pinene (55%) present in the EO [6]. Therefore, in addition to the antagonism in the grape seed mix, the co-oxidizing activity of α-pinene further decreases the antioxidant activity. 

EOs enriched with the green tea mix (Table 3) showed the lowest IC_50_ values among all enrichments and moderate to high ORAC values (6208.5–13,681.0 μmol TE/g sample). Pimento berry (HE-PIM-01)/green tea mix showed the highest overall antioxidant capacity (IC_50_ = 0.009 mg/mg DPPH; ORAC Value = 13,681.0 μmol TE/g sample). This pair’s synergistic effects and low IC_50_ value could be due to the contributions of eugenol and the EO’s minor components such as methyleugenol. Since clove oil (HE-CLO-01) contains more eugenol (93.5% versus 86.4%), its lower activity confirms that other compounds are influencing pimento berry’s (HE-PIM-01) activity [6]. The green tea mix showed low, antagonistic ORAC values when enriching oregano (HE-ORI-03) and yellow sage (HE-SAU-01-02), due to the lack of phenols in their chemical profiles and/or the presence of pro-oxidants, which can generate reactive species (hydrogen peroxide) that quench the fluorescein and decrease the antioxidant capacity [21].

EOs enriched with the apple polyphenol mix (Table 3) did not show antagonistic effects in the DPPH assay, however, there was antagonism in the ORAC assay when enriching oregano (HE-ORI-03, 1151.6 μmol TE/g sample), white thyme (HE-THY-03, 1156.4 μmol TE/g sample), and yellow sage (HE-SAU-01-02, 601.6 μmol TE/g sample), with the last enrichment exhibiting the poorest activity. This antagonism could have arisen from the oxidation of the primary antioxidant(s) by the synergist(s) or the regeneration of the latter by the former [7]. Since yellow sage (HE-SAU-01-02) exhibited low, antagonistic ORAC values when paired with chlorogenic acid and rutin (Table 1), perhaps both enrichments resulted in the oxidation/regeneration, and by combining them, any trace of effective antioxidant was practically lost. Additionally, the possible presence of pro-oxidant monoterpenes in yellow sage could have contributed to the antagonism. The enrichment of clove (HE-CLO-01) with the apple mix showed synergistic effects in both assays (IC_50_ = 0.013 mg/mg DPPH; ORAC Value = 12,720.5 μmol TE/g sample), whereas enrichments of pimento berry (HE-PIM-01) (IC_50_ = 0.104 mg/mg DPPH; ORAC value = 11,740.0 μmol TE/g sample) and Ceylon cinnamon (HE-CAN-04) (IC_50_ = 0.116 mg/mg DPPH; ORAC Value = 11,338.5 μmol TE/g sample) showed additive/synergistic and synergistic/additive effects in the DPPH/ORAC assays, respectively. When paired with the apple mix, these EOs exhibited ORAC values more than ten times greater than the other EOs, due to synergistic interactions with their eugenol. Indeed, the ORAC values increase with the EOs’ increasing eugenol contents (clove, HE-CLO-01, 93.5% > pimento berry, HE-PIM-01, 86.4% > Ceylon cinnamon, HE-CAN-04, 84.6%) [6].

All EOs paired with the rosemary polyphenol mix (Table 3) did not exhibit any antagonism in the DPPH assay. However, this enrichment yielded some of the highest IC_50_ values, although all were synergistic. The high absolute values of the IC_50_ of these enrichments could be attributed to the presence of *p*-coumaric acid (80% of the mix), which was shown to have a poor antioxidant activity on its own (Table 1), due to the absence of a catechol group in its structure. In the ORAC assay, enrichments of all EOs exhibited synergistic and additive effects, except those with yellow sage (HE-SAU-01-02, 8620.0 μmol TE/g sample). Pimento berry (HE-PIM-01) and Ceylon cinnamon (HE-CAN-04) EOs exhibited greater ORAC values (16,148.0 μmol TE/g sample, 18,477.00 μmol TE/g sample, respectively) than EOs lacking eugenol. However, the enrichment of clove EO (ORAC value = 12,482.5 μmol TE/g sample) resulted in additive effects, despite having a greater amount of eugenol (93.5% versus 86.4%, 84.6%, respectively), suggesting that interactions with minor oxygenated compounds, such as alcohols, contributed to their synergistic effects. 

### 3.3. Antioxidant Capacity of EOs Enriched with Crude Extracts

The enrichment of all EOs with all plant extracts (Table 3) yielded synergistic effects in the DPPH assay, except pimento berry (HE-PIM-01)/rosemary extract, which exhibited additive effects. No antagonistic effects were observed in this assay. In the ORAC assay, less antagonistic effects were observed in comparison to enrichments with polyphenol mixes (Table 3) (four versus seven, respectively). Additionally, there were increased additive effects in this assay and an equal number of synergistic effects in the same comparison. 

Enrichment of all oils, except pimento berry (HE-PIM-01) and Ceylon cinnamon (HE-CAN-04), with the grape seed extract (EX-RAI-01) greatly improved the antioxidant capacities of the EOs in both assays, in comparison to their corresponding values with the polyphenol mix. When clove EO (HE-ORI-03) was enriched with the grape seed polyphenol mix (Table 3), it exhibited antagonistic effects; however, when enriched with the extract, this pair showed synergistic effects (Table 3), suggesting that interactions with the extract’s minor components contributed to the antioxidant capacity. In [22], they found that a mix of the major compounds in grape seed skins, including epicatechin, catechin, and gallic acid, contributed to less than 26% of their antioxidant properties, confirming the positive role of other minor components. The ORAC values of pimento berry (HE-PIM-01) and Ceylon cinnamon (HE-CAN-04) remained similar to their enrichments with the polyphenol mix and exhibited antagonistic effects, indicating that the major polyphenols were the main compounds driving the synergy. 

Enrichment of all oils with the green tea extract (EX-THE-01) increased their antioxidant capacity, especially pimento berry’s (HE-PIM-01) ORAC value (39,921.5 μmol TE/g sample), which showed synergistic effects. No antagonism was observed in both assays: all enrichments in the DPPH assay were synergistic, whereas those in the ORAC assay were additive, except with pimento berry (HE-PIM-01). The ORAC values of the EOs nearly doubled, suggesting that interactions with the major compounds contributed to half of the effects and that the extract’s minor polyphenols contributed to the other half. Green tea is known to have a very high antioxidant activity thanks to its flavonoids, catechins, gallic acids, and the like [23]. With an abundance of these compounds, combining EOs with green tea could result in the regeneration of many powerful antioxidants and stable phenoxyl radicals. 

Compared to the apple mix enrichments, enrichments with the crude apple extract (EX-POM-04) improved the IC_50_ and ORAC values of all the EOs, except the IC_50_ of clove (HE-CLO-03) (0.041 mg/mg DPPH). No antagonistic effects were observed in both assays. Enrichments that once were antagonistic between EOs and the apple polyphenol mix were then additive (oregano, HE-ORI-03; yellow sage, HE-SAU-01-02) or synergistic (white thyme, HE-THY-03) when enriched with the plant extract. Therefore, the minor components of the plant extract contribute positively to the overall antioxidant activity of the enrichments. Furthermore, all synergistic combinations included EOs with phenols (Table S1), whereas those that exhibited additive effects included EOs lacking phenols (Table S1). All enrichments in the DPPH assay were synergistic, suggesting that both major and minor components of plant extract interact synergistically with all EOs, regardless of the presence of phenols.

Rosemary extract (EX-ROM-04) enrichments showed the lowest overall improvement in absolute antioxidant capacity. Enriching with the extract improved the IC_50_ compared to the polyphenol mix and showed synergistic effects across all enrichments, except that of pimento berry (HE-PIM-01, 0.126 mg/mg DPPH), which exhibited additive effects. In the ORAC assay, there were antagonistic effects when enriching yellow sage (HE-SAU-01-02) and oregano (HE-ORI-03) with the extract. Therefore, the antagonistic effects between yellow sage (HE-SAU-01-02) and the rosemary polyphenol mix had carried over and interactions with the minor components did not overcome the pre-existing antagonism. Additionally, the synergy that was previously present between oregano (HE-ORI-03)/rosemary mix was then lost when enriched with the crude extract. This reveals that the minor components of rosemary extract (EX-ROM-04) interact poorly with EOs lacking phenols in the ORAC assay. The enrichment of clove EO (HE-CLO-01) remained synergistic, albeit with a lower ORAC value than that with the polyphenol mix. Enrichment of pimento berry (HE-PIM-01) and Ceylon cinnamon (HE-CAN-04) remained synergistic, though enrichments with the extract resulted in lower ORAC values. This suggests that the major compounds of rosemary extract were mostly responsible for their synergistic effects. 

### 3.4. Prediction of the Effects of EO Enrichment

**Figure 1:**
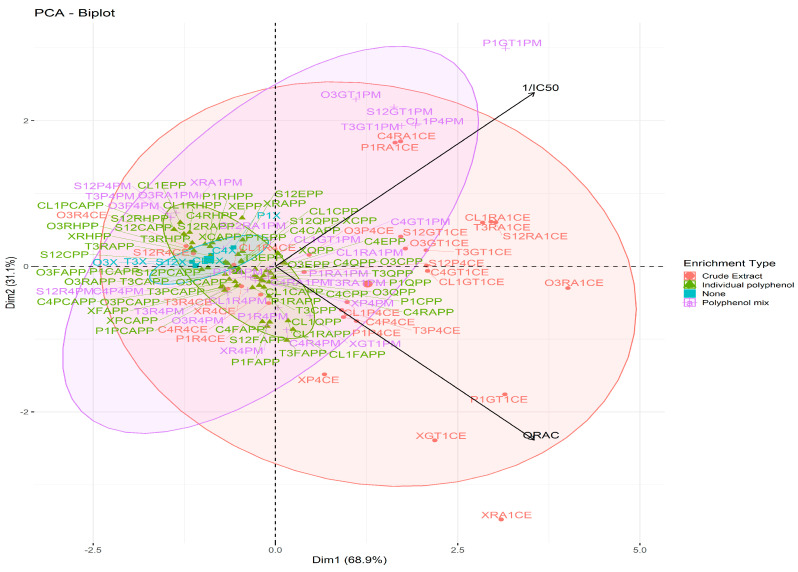
Principal component analysis of non-enriched (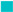) and enriched EOs with individual polyphenols (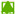), polyphenol mixes (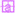), and crude plant extracts (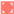).

Overall, each enrichment step behaved differently in both assays, as shown in the principal component analysis (PCA) (Figure 1). Unenriched and enriched oils with polyphenol mixes followed the 1/IC_50_ vector, indicating that they were well differentiated in the DPPH assay. The ORAC values of the latter did not vary much, hence the lack of dispersion along the ORAC vector. Therefore, to assess the contribution of each polyphenol mix and their interactions with the EOs, the DPPH assay could be considered. Oils enriched with individual polyphenols followed the ORAC vector, as their IC_50_ values were not dispersed enough to be differentiated between each other. Finally, though all oil/crude extract enrichments were loosely dispersed from each other, enrichments with the same extract clustered uniformly along the ORAC vector, with a few outliers (oil/green tea extract enrichments). This, therefore, suggests that these enrichments are loosely differentiated by the ORAC assay.

**Figure 2:**
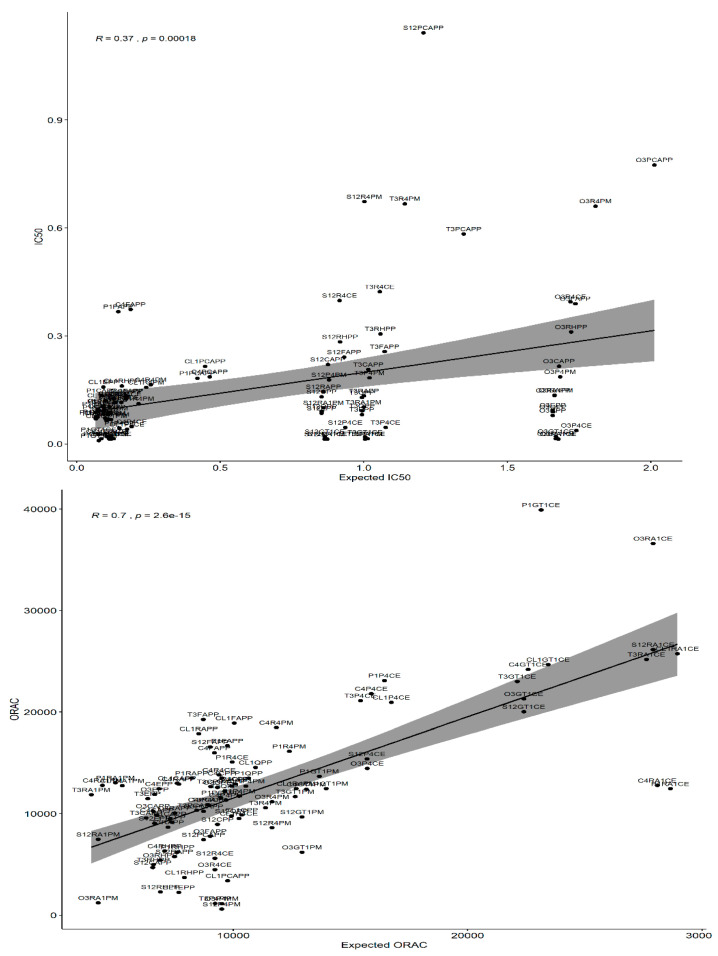
Correlations between the observed and expected IC_50_ and ORAC.

Figure 2 shows the correlations between the observed and expected IC_50_ and ORAC values. There is a weak to moderate correlation between the expected and observed IC_50_ (R^2^ = 0.37). The difference between the two is more pronounced in the high IC_50_ value range. Additionally, the correlation is not proportional. The following model can explain 48% of the variability of observed IC_50_ (R^2^ = 0.48):

Predicted IC_50_ = −0.0007 + 0.09 × (Oil IC_50_) + 1.4 × (Enrichment IC_50_)

In contrast, there was a moderate to strong correlation between the expected and observed ORAC values (R^2^ = 0.7). The variability was mainly due to the synergistic and antagonistic effects observed upon enrichment with plant extracts than with individual polyphenols and polyphenol mixes. The following model can explain 50% of the variability of the observed IC_50_ (R^2^ = 0.48).

Predicted ORAC = −1014 + 2.073 × (Oil ORAC) + 0.79 × (Enrichment ORAC).

## 4. Conclusions

The investigation of the interactions between selected EOs enriched with polyphenols, their mixtures, and plant extracts was conducted using the DPPH and ORAC assays. No direct correlation was found between the IC_50_ and ORAC values, due to solvent effects and the presence of pro-oxidants. Enrichments with polyphenol mixtures and plant extracts eliminated antagonistic effects in the DPPH assay. Furthermore, the latter enrichment showed a decrease in antagonistic effects in the ORAC assay, suggesting that interactions with the minor compounds of extracts, such as green tea (EX-THE-01) and apple (EX-POM-04) extracts, contributed positively to their activity. Such interactions are advantageous, as lower EO quantities would be necessary to increase product quality. Predictive models were developed to explain the variability in the IC_50_ and ORAC values. In future work, the use of additional antioxidant activity assays with different mechanisms, including reducing power, metal chelation, and others, would broaden the potential applications of the identified synergistic multi-oxidant systems.

## Figures and Tables

**Table 1:** Antioxidant capacity (ORAC and DPPH assays) of essential oils enriched with individual polyphenols.

Enrichment	Oil ID ^a^	IC_50_ (mg Oil + Polyphenol/mg DPPH)	Effect ^b^	ORAC Value (µmol TE/g Oil + Polyphenol)	Effect ^b^
Rutin Hydrate	N/A	0.178 ± 0.003 ^c^	N/A	7075.9 ± 2.1 ^c^	N/A
	HE-CLO-01	0.161 ± 0.004	+	3716.9 ± 4.3	−
	HE-PIM-01	0.114 ± 0.005	+	6227.0 ± 5.9	−
	HE-ORI-03	0.311 ± 0.000	+ + +	5427.5 ± 6.2	−
	HE-THY-03	0.306 ± 0.000	+ + +	4986.5 ± 2.7	−
	HE-SAU-01-02	0.284 ± 0.001	+ + +	2298.5 ± 1.2	−
	HE-CAN-04	0.133 ± 0.007	+ + +	6329.5 ± 3.2	+
Rosmarinic Acid	N/A	0.063 ± 0.001	N/A	8296.2 ± 1.9	N/A
	HE-CLO-01	0.121 ± 0.004	−	17,879.5 ± 4.1	+ + +
	HE-PIM-01	0.097 ± 0.001	−	13,511.0 ± 3.8	+ + +
	HE-ORI-03	0.135 ± 0.002	+ + +	10,067.0 ± 1.2	+ + +
	HE-THY-03	0.134 ± 0.002	+ + +	8680.0 ± 1.4	+ + +
	HE-SAU-01-02	0.145 ± 0.001	+ + +	5772.0 ± 1.8	−
	HE-CAN-04	0.089 ± 0.003	+ + +	12,916.0 ± 3.9	+ + +
Quercetin	N/A	0.051 ± 0.002	N/A	13,143.0 ± 0.5	N/A
	HE-CLO-01	0.106 ± 0.004	+	14,567.0 ± 1.9	+ + +
	HE-PIM-01	0.070 ± 0.002	+	13,502.5 ± 1.9	+ + +
	HE-ORI-03	0.089 ± 0.002	+ + +	12,714.0 ± 0.9	+ + +
	HE-THY-03	0.092 ± 0.002	+ + +	12,228.5 ± 1.8	+ + +
	HE-SAU-01-02	0.091 ± 0.002	+ + +	9776.0 ± 1.9	+
	HE-CAN-04	0.068 ± 0.001	+ + +	12,921.5 ± 1.7	+ + +
p-Coumaric acid	N/A	0.759 ± 0.010	N/A	10,762.0 ± 3.2	N/A
	HE-CLO-01	0.215 ± 0.003	+ + +	3400.0 ± 4.8	−
	HE-PIM-01	0.182 ± 0.002	+ + +	11,588.0 ± 4.5	+ + +
	HE-ORI-03	0.775 ± 0.007	+ + +	10,242.5 ± 0.9	+
	HE-THY-03	0.583 ± 0.006	+ + +	10,349.0 ± 2.1	+ + +
	HE-SAU-01-02	1.142 ± 0.005	+	7451.0 ± 2.6	−
	HE-CAN-04	0.186 ± 0.006	+ + +	10,892.5 ± 5.3	+ + +
Epicatechin	N/A	0.049 ± 0.003	N/A	6593.0 ± 1.0	N/A
	HE-CLO-01	0.158 ± 0.006	−	2252.4 ± 2.6	−
	HE-PIM-01	0.075 ± 0.002	+	9170.5 ± 2.1	+ + +
	HE-ORI-03	0.094 ± 0.006	+ + +	11,907.5 ± 1.6	+ + +
	HE-THY-03	0.082 ± 0.001	+ + +	11,489.0 ± 0.7	+ + +
	HE-SAU-01-02	0.085 ± 0.006	+ + +	9058.5 ± 2.8	+ + +
	HE-CAN-04	0.069 ± 0.003	+ + +	12,459.0 ± 2.4	+ + +
Chlorogenic Acid	N/A	0.093 ± 0.001	N/A	6438.2 ± 2.7	N/A
	HE-CLO-01	0.103 ± 0.000	+	13,014.5 ± 3.2	+ + +
	HE-PIM-01	0.134 ± 0.004	−	9497.5 ± 3.1	+ + +
	HE-ORI-03	0.216 ± 0.001	+ + +	10,272.0 ± 2.7	+ + +
	HE-THY-03	0.207 ± 0.003	+ + +	9599.5 ± 2.8	+ + +
	HE-SAU-01-02	0.221 ± 0.001	+ + +	4709.4 ± 2.3	−
	HE-CAN-04	0.126 ± 0.002	+	9870.0 ± 3.2	+ + +
Catechin	N/A	0.049 ± 0.002	N/A	11,950.0 ± 6.9	N/A
	HE-CLO-01	0.092 ± 0.004	+ + +	9887.0 ± 3.0	+
	HE-PIM-01	0.088 ± 0.002	−	12,937.0 ± 3.0	+ + +
	HE-ORI-03	0.079 ± 0.001	+ + +	12,603.0 ± 1.5	+ + +
	HE-THY-03	0.129 ± 0.005	+ + +	12,669.5 ± 3.4	+ + +
	HE-SAU-01-02	0.131 ± 0.004	+ + +	8947.0 ± 2.3	+
	HE-CAN-04	0.081 ± 0.003	+ + +	13,511.0 ± 1.9	+ + +
Ferulic Acid	N/A	0.207 ± 0.001	N/A	18,915.0 ± 0.1	N/A
	HE-CLO-01	0.134 ± 0.001	+ + +	18,915.0 ± 1.7	+ + +
	HE-PIM-01	0.367 ± 0.004	−	16,703.5 ± 2.3	+ + +
	HE-ORI-03	0.389 ± 0.001	+ + +	7787.5 ± 0.5	−
	HE-THY-03	0.256 ± 0.003	+ + +	19,273.5 ± 0.4	+ + +
	HE-SAU-01-02	0.241 ± 0.001	+ + +	16,581.0 ± 0.4	+ + +
	HE-CAN-04	0.374 ± 0.002	−	16,004.0 ± 0.3	+ + +

^a^ HE-CLO-01: Clove EO; HE-PIM-01: Pimento Berry EO; HE-ORI-03: Oregano EO; HE-THY-03: Thyme EO; HE-SAU-01-02: Yellow Sage EO; HE-CAN-04: Cinnamon EO. ^b^ Interaction effects were identified to be antagonistic (−), additive (+), or synergistic (+ + +) when the antioxidant capacity of mixture was respectively less than, equal to, or greater than the antioxidant capacity of the sum of its individual compounds (expected value). Expected values were calculated based on the sum of the antioxidant activities of individual compounds. ^c^ Standard deviations.

**Table 2:** Combinatorial effects of polyphenol mixtures (ORAC and DPPH assays).

Polyphenol Mix ^a^	Expected IC_5_0 (mg/mg DPPH)	Measured IC_50_ (mg/mg DPPH)	Effect ^b^	Expected ORAC (µmol TE/g Mix)	Measured ORAC (µmol TE/g Mix)	Effect ^b^
EX-RAI-01	0.049	0.062	−	9170.9	1772.5	−
EX-THE-01	0.054	0.071	−	7732.5	19,167.0	+ + +
EX-POM-04	0.091	0.101	−	7288.5	12,318.0	+ + +
EX-ROM-04	0.618	0.348	+ + +	10,263.4	16,611.0	+ + +

^a^ EX-RAI-01: Grape seed extract; EX-THE-01: Green tea extract; EX-POM-04: Apple extract; EX-ROM-04: Rosemary extract. Polyphenol mixes consist of the major compounds present in their respective plant extracts. Expected values were calculated based on the sum of the antioxidant activities of individual compounds. See materials and methods for proportions. ^b^ Interaction effects were identified to be antagonistic (−), additive (+), or synergistic (+ + +) when the antioxidant capacity of the mix was respectively less than, equal to, or greater than the antioxidant capacity of the sum of its individual compounds (expected value).

**Table 3:** Antioxidant capacity of EOs (ORAC and DPPH assays) enriched with polyphenol mixtures and pure plant extracts.

		Polyphenol Mix				Crude Plant Extract			
Enrichment ^a^	Essential Oil ID ^b^	IC_50_ (mg oil/mg DPPH)	Effect ^c^	ORAC Value (μmol TE/g Sample)	Effect	IC_50_ (mg oil/mg DPPH)	Effect	ORAC Value (μmol TE/g Sample)	Effect
EX-RAI-01	N/A	0.062 ± 0.005 ^d^	N/A	1772.5 ± 0.9 ^d^	N/A	0.089 ± 0.004 ^d^	N/A	49,119.0 ± 0.6 ^d^	N/A
	HE-CLO-01	0.0713 ± 0.002	+ + +	12,764.5 ± 1.8	+ + +	0.014 ± 0.000	+ + +	25,758.5 ± 3.4	+
	HE-PIM-01	0.0744 ± 0.003	+	13,058.0 ± 1.6	+ + +	0.015 ± 0.000	+ + +	12,468.5 ± 3.4	−
	HE-ORI-03	0.1348 ± 0.006	+ + +	1219.4 ± 0.4	−	0.013 ± 0.000	+ + +	36,607.5 ± 1.5	+ + +
	HE-THY-03	0.1022 ± 0.001	+ + +	11,870.0 ± 0.7	+ + +	0.014 ± 0.000	+ + +	25,178.0 ± 2.4	+
	HE-SAU-01-02	0.1006 ± 0.001	+ + +	7483.0 ± 1.4	+ + +	0.014 ± 0.000	+ + +	26,152.5 ± 1.4	+
	HE-CAN-04	0.085 ± 0.002	+ + +	12,796.5 ± 3.7	+ + +	0.014 ± 0.000	+ + +	12,790.5 ± 2.4	−
EX-THE-01	N/A	0.0714 ± 0.005	N/A	19,167.0 ± 1.5	N/A	0.072 ± 0.003	N/A	38,095.0 ± 1.7	N/A
	HE-CLO-01	0.064 ± 0.000	+ + +	12,473.5 ± 2.4	+ +	0.020 ± 0.000	+ + +	24,684.0 ± 1.6	+
	HE-PIM-01	0.009 ± 0.000	+ + +	13,681.0 ± 1.8	+ +	0.026 ± 0.000	+ + +	39,921.5 ± 2.9	+ + +
	HE-ORI-03	0.014 ± 0.000	+ + +	6207.5 ± 0.8	−	0.020 ± 0.021	+ + +	21,298.5 ± 6.6	+
	HE-THY-03	0.014 ± 0.000	+ + +	11,692.5 ± 1.7	+ +	0.019 ± 0.000	+ + +	23,003.0 ± 2.9	+
	HE-SAU-01-02	0.013 ± 0.000	+ + +	9685.5 ± 0.5	−	0.020 ± 0.001	+ + +	20,030.0 ± 1.9	+
	HE-CAN-04	0.068 ± 0.000	+ + +	12,413.0 ± 4.8	+ +	0.020 ± 0.000	+ + +	24,199.0 ± 2.4	+
EX-POM-04	N/A	0.101 ± 0.001	N/A	12,318.0 ± 2.0	N/A	0.215 ± 0.013	N/A	24,721.0 ± 3.5	N/A
	HE-CLO-01	0.013 ± 0.004	+ + +	12,720.5 ± 3.0	+ + +	0.041 ± 0.000	+ + +	20,954.0 ± 1.7	+ + +
	HE-PIM-01	0.104 ± 0.003	+	11,740.0 ± 3.4	+	0.044 ± 0.001	+ + +	23,100.0 ± 1.8	+ + +
	HE-ORI-03	0.186 ± 0.001	+ + +	1151.6 ± 1.1	−	0.038 ± 0.000	+ + +	14,475.0 ± 0.9	+
	HE-THY-03	0.184 ± 0.001	+ + +	1156.4 ± 1.6	−	0.046 ± 0.000	+ + +	21,128.0 ± 1.1	+ + +
	HE-SAU-01-02	0.178 ± 0.001	+ + +	601.6 ± 0.7	−	0.045 ± 0.000	+ + +	15,410.5 ± 1.2	+
	HE-CAN-04	0.116 ± 0.004	+ + +	11,338.5 ± 2.4	+ +	0.048 ± 0.001	+ + +	21,813.5 ± 1.9	+ + +
EX-ROM-04	N/A	0.348 ± 0.003	N/A	16,611.0 ± 1.6	N/A	0.174 ± 0.008	N/A	11,729.0 ± 1.3	N/A
	HE-CLO-01	0.157 ± 0.004	+ + +	12,481.5 ± 4.3	+	0.115 ± 0.004	+ + +	9529.5 ± 2.2	+
	HE-PIM-01	0.112 ± 0.006	+ + +	16,148.0 ± 0.9	+ + +	0.126 ± 0.003	+	15,089.0 ± 2.4	+ + +
	HE-ORI-03	0.660 ± 0.015	+ + +	11,193.5 ± 2.6	+	0.395 ± 0.004	+ + +	4487.3 ± 0.3	−
	HE-THY-03	0.667 ± 0.012	+ + +	10,590.0 ± 3.8	+	0.423 ± 0.007	+ + +	10,893.5 ± 1.8	+ + +
	HE-SAU-01-02	0.673 ± 0.009	+ + +	8620.0 ± 1.2	−	0.398 ± 0.005	+ + +	5605.5 ± 1.5	−
	HE-CAN-04	0.164 ± 0.001	+ + +	18,477.0 ± 0.9	+ + +	0.124 ± 0.003	+ + +	13,828.0 ± 2.1	+ + +

^a^ EX-RAI-01: Grape seed extract; EX-THE-01: Green tea extract; EX-POM-04: Apple extract; EX-ROM-04: Rosemary extract ^b^ HE-CLO-01: Clove EO; HE-PIM-01: Pimento Berry EO; HE-ORI-03: Oregano EO; HE-THY-03: Thyme EO; HE-SAU-01-02: Yellow Sage EO; HE-CAN-04: Cinnamon EO ^c^ Interaction effects were identified to be antagonistic (−), additive (+), or synergistic (+ + +) when the antioxidant capacity of the enriched mix or extract was respectively less than, equal to, or greater than the antioxidant capacity of the sum of its individual compounds (expected value). Expected values were calculated based on the sum of the antioxidant activities of individual compounds. ^d^ Standard deviations.

## Data Availability

The data presented in this study are available on request from the corresponding author.

## Supplementary Materials

The following supporting information can be downloaded at: https://www.mdpi.com/article/10.3390/antiox12020486/s1, Supplementary Table S1: List of EOs and their origins; Supplementary Table S2: Preparation of multi-component polyphenol mixes as analogues of major compounds present in selected plant extracts.
