# Supplementary material for: Combinatorial Interactions of Essential Oils Enriched with Individual Polyphenols, Polyphenol Mixes, and Plant Extracts: Multi-Antioxidant Systems
Source: Antioxidants (Basel). 2023 Feb 15;12(2):486. doi: 10.3390/antiox12020486 (PMC9952583; doi:10.3390/antiox12020486)
Supplement: Supplementary file 1 [file antioxidants-12-00486-s001.zip › antioxidants-2172855-supplementary.pdf]

Supplementary Table S1

List of EOs and their origins

ID	EO[a]	Latin name	Distilled part	Origin	Supplier	Major Families of compounds
HE-PIM-01	Pimento Berry	*Pimenta officinalis Lindley*	Seed	Unknown	BSA	Phenol, Sesquiterpene
HE-CAN-04	Ceylon Cinnamon	*Cinnamomum verum (zeylanicum)*	Leaf	Unknown	BSA	Phenol, Esters, Alcohol
HE-CLO-01	Clove	*Eugenia caryophyllus*	Flower bud	Madagascar	Aliksir	Phenol, Sesquiterpene, Monoterpene
HE-SAU-01-02	Sage	*Salvia officinalis L.*	Aerial part	Unknown	BSA	Monoterpene/ketone
HE-THY-03	White thyme	*Thymus zygis*	Leaves	France	Novotaste	Phenol, Monoterpene, Alcohol
HE-ORI-03	Oregano	*Origanum vulgare v. kaliteria*	Aerial part	Hungary	Novotaste	Monoterpene, Alcohol

[a]*EO*: Essential Oil

Supplementary Table S2

Preparation of multi-component polyphenol mixes as analogues of major compounds present in selected plant extracts.

Extract	Country of Origin	Supplier	ID	Polyphenol	% (w/w) Total Polyphenols[a]
Grape Seed	China	New Directions	EX-RAI-01	Catechin	48
				Epicatechin	52
Green Tea	China	New Directions	EX-THE-01	Chlorogenic Acid	1.1
				Catechin	21
				Epicatechin	76
				p-Coumaric Acid	0.4
				Rutin Hydrate	1.2
Apple	-	Diana Foods	EX-POM-04	Chlorogenic Acid	81
				Epicatechin	2.1
				Rutin Hydrate	4.3
				Quercetin	12
Rosemary	-	BSA	EX-ROM-04	p-Coumaric Acid	80
				Rosmarinic Acid	20

[a]Liquid chromatography/mass spectrometry (LC/MS) analysis of the extracts was carried using Agilent 6545 Q-TOF and ZORBAX Rapid Resolution High Definition column. The mass spectrometry measurements were conducted in both positive and negative mode using a Dual AJS ESI ion source.
